# Stakeholder engagement in research on quality of life and palliative care for brain tumors: a qualitative analysis of #BTSM and #HPM tweet chats

**DOI:** 10.1093/nop/npaa043

**Published:** 2020-07-29

**Authors:** Liz Salmi, Hillary D Lum, Adam Hayden, Maija Reblin, Shirley Otis-Green, Grace Venechuk, Megan A Morris, Megan Griff, Bethany M Kwan

**Affiliations:** 1 Department of General Medicine and Primary Care, Beth Israel Deaconess Medical Center, Boston, Massachusetts; 2 VA Geriatric Research Education and Clinical Center, Rocky Mountain Regional VA Medical Center, Aurora, Colorado; 3 Division of Geriatric Medicine, University of Colorado School of Medicine, University of Colorado Anschutz Medical Campus, Aurora, Colorado; 4 Philosophy, Indiana University-Purdue University, Indianapolis, Indiana; 5 Department of Health Outcomes & Behavior, Moffitt Cancer Center, Tampa, Florida; 6 Collaborative Caring, Toluca Lake, California; 7 Adult and Child Consortium for Health Outcomes Research and Delivery Science, University of Colorado Anschutz Medical Campus, Aurora, Colorado; 8 Department of Family Medicine, University of Colorado School of Medicine, University of Colorado Anschutz Medical Campus, Aurora, Colorado

**Keywords:** brain tumors, palliative care, quality of life, social media, stakeholder engagement

## Abstract

**Background:**

Research is needed to inform palliative care models that address the full spectrum of quality of life (QoL) needs for brain tumor patients and care partners. Stakeholder engagement in research can inform research priorities; engagement via social media can complement stakeholder panels. The purpose of this paper is to describe the use of Twitter to complement in-person stakeholder engagement, and report emergent themes from qualitative analysis of tweet chats on QoL needs and palliative care opportunities for brain tumor patients.

**Methods:**

The Brain Cancer Quality of Life Collaborative engaged brain tumor (#BTSM) and palliative medicine (#HPM) stakeholder communities via Twitter using tweet chats. The #BTSM chat focused on defining and communicating about QoL among brain tumor patients. The #HPM chat discussed communication about palliative care for those facing neurological conditions. Qualitative content analysis was used to identify tweet chat themes.

**Results:**

Analysis showed QoL for brain tumor patients and care partners includes psychosocial, physical, and cognitive concerns. Distressing concerns included behavioral changes, grief over loss of identity, changes in relationships, depression, and anxiety. Patients appreciated when providers discussed QoL early in treatment, and emphasized the need for care partner support. Communication about QoL and palliative care rely on relationships to meet evolving patient needs.

**Conclusions:**

In addition to providing neurological and symptom management, specialized palliative care for brain tumor patients may address unmet patient and care partner psychosocial and informational needs. Stakeholder engagement using Twitter proved useful for informing research priorities and understanding stakeholder perspectives on QoL and palliative care.

The 5-year average survival rate for all types of brain tumors is 36%; the lowest survival rate is at 6.8% for glioblastoma, the most common malignant brain tumor.^1^ People with primary brain tumors (henceforth “patients”) and family members and friends who care for them (henceforth “care partners”) face many physical, psychosocial, and spiritual challenges affecting quality of life (QoL).^2^ Symptoms associated with these diseases and treatments can be a great burden both to patients and their care partners.^3^ Patients may experience significant physical debilitation, cognitive decline, distress, anxiety, and a range of personality and behavior changes,^2,4,5^ while care partners simultaneously struggle to manage their own emotional distress with a patient’s rapidly changing needs.^3,6^ The multidimensional QoL concerns commonly faced by brain tumor patients and care partners may be addressed by integrating palliative care simultaneously with disease-modifying interventions.^7^

Palliative care is specialized interprofessional care that focuses on improving the QoL of patients and care partners facing the multidimensional challenges associated with serious illness.^8^ Best oncology practice integrates palliative care simultaneously with disease-modifying interventions.^7^ Evidence from non–brain tumor populations suggests offering palliative care at the point of diagnosis of a serious illness—with standard of care—may improve QoL, symptom management, decrease unwanted invasive interventions at the end of life,^9^ and potentially improve overall survival.^10^

Despite the potential QoL benefits provided by palliative care, and a clear need in this population, palliative care services are often underused in brain tumor settings, particularly early in the care trajectory when they may be most beneficial.^11–13^ This lack of utilization is likely at least in part due to common barriers to utilization of palliative care (eg, lack of palliative care training, lack of awareness of the literature supporting early integration of palliative care with oncology patients),^14–16^ with potential difficulties for clinicians in neuro-oncology including *when* and *how* to offer palliative care in a way that is sensitive to the QoL concerns and preferences of patients and their care partners.^17^ Furthermore, there is a need for stronger evidence on QoL assessments and palliative care that meets the needs and preferences specific to brain tumor patients and their care partners.^18^

One way to better understand patient and care partner perspectives on opportunities for research in palliative care specific to care of individuals with brain tumors is through stakeholder engagement. Stakeholders in research can include patients, care partners, health care providers and system leaders, policy makers, and others.^19^*Stakeholder engagement* in research refers to “a bi-directional relationship between the stakeholder and researcher that results in informed decision-making about the selection, conduct, and use of research.” ^19^ A variety of methods exist for stakeholder engagement in research, such as stakeholder panels, focus groups, key informant interviews, and town halls.^20,21^ Stakeholder panels are a common engagement method, consisting of representatives selected from relevant communities to partner on setting research priorities, as well as research conduct and evidence dissemination. Stakeholder panels tend to engage patients and other stakeholders as co-equal collaborative partners in research—which is an important goal for authentic engagement.^22^ However, stakeholder panels may be at risk of bias toward the perspectives of the individual panel members. Complementary methods for engaging and eliciting stakeholder perspectives beyond the panel may be warranted. For instance, rare disease research increasingly harnesses the power of social media and online networks to enable more diverse groups of patients to engage as partners in research.^23^

The Brain Cancer Quality of Life Collaborative (BCQoLC; www.braincancerqol.org) is composed of people with brain tumors, care partners, researchers, palliative care and neuro-oncology clinicians, advocacy group leaders, and payer representatives that was funded with support from the Patient-Centered Outcomes Research Institute (PCORI). The BCQoLC uses multiple engagement strategies to establish research priorities related to QoL and palliative care for people with brain tumors, including a multistakeholder panel and design-thinking workshops.^24^ At its founding, the BCQoLC endorsed a definition of QoL as having 4 domains: physical, psychological, social, and spiritual. A design-thinking workshop in which the panel participated posed the question, “How might we learn how to best deliver palliative care to brain cancer patients and their care partners?” As a result, the stakeholders developed a prototype concept for a patient-centered palliative care model for brain cancer that would leverage palliative care teams and services designed to improve functioning and relieve suffering across all 4 domains of QoL.

To understand stakeholder perspectives beyond the panel and to validate ideas that emerged from design thinking, we used “tweet chats” to engage existing Twitter communities in exploration of these topics. A *tweet chat* is a live discussion on Twitter—set at a specific date/time—in which Twitter users participate by including an agreed-on hashtag (eg, #BTSM for brain tumor social media, or #HPM for hospice and palliative medicine) to aggregate conversations. In health care, tweet chats can range in purpose from discussions of specific topics to disseminating information or resources.^25^ Recent precedent shows data from tweet chats (such as chat transcripts) can be usefully analyzed in a variety of ways, including sentiment analysis and qualitative thematic analysis.^25–27^

The purpose of this paper is to: 1) describe the use of Twitter to engage brain tumor and palliative care stakeholder communities in research on QoL; and 2) to present emergent themes from qualitative analysis of 2 tweet chats on QoL needs for people with brain tumors and experiences discussing QoL and palliative care with health care providers, respectively.

## Methods

### Design

We conducted 2 separate hour-long tweet chats with 2 well-established Twitter groups in spring 2018: one with the #BTSM community and one with the #HPM community. We employed qualitative content analysis^28^ to inductively identify themes from the transcripts of the 2 chats. The overarching study question and design were informed by shared decision making among members of the BCQoLC. Having a better understanding of the patient, care partner, and palliative care clinician views on QoL, communication preferences, and how to optimize palliative care services were ranked as a high priority for the BCQoLC.

### Setting

The tweet chats were conducted in collaboration with #BTSM and #HPM communities. We coordinated with both of these hashtag communities in the effort to reflect the stakeholder perspectives represented by BCQoLC. #BTSM and #HPM both host monthly tweet chats at a regular date and time and their participants are accustomed to the process. #BTSM hosts a 1-hour live tweet chat the first Sunday of each month at 6 pm Pacific time (PT) and is facilitated by the account @BTSMchat. #BTSM was adopted by the American Society of Clinical Oncology as part of the Cancer Tag Ontology for Twitter,^29^ and guidelines have been introduced for neuro-oncology professionals interacting with patients on social media.^30^ The #HPM tweet chat takes place the last Wednesday of each month at 6 pm PT and is facilitated by the account @HPMchat. Like many online spaces, “healthcare Twitter” has its own culture, which includes discussing specific topics, disseminating research, sharing information, and advocacy.^31^ #BTSM and #HPM are among many health care hashtag communities.^32^

For this study, the #BTSM tweet chat discussed defining and communicating about QoL for people with brain tumors. The #HPM chat discussed communication about palliative care for people experiencing serious neurological or cognitive conditions. The #HPM chat focused on communication techniques and challenges and discussing palliative care for people with neurological conditions, which includes those with brain tumors.

Given that participation in and data from tweet chats are publicly available and the purpose was for stakeholder engagement, the project was determined to be Non-Human Subjects Research by the (Colorado Multiple Institution Review Board).

### Participants

#BTSM and #HPM both hold tweet chats on a monthly basis and are open to the public. Those who participate in discussions using these hashtags are Twitter users who self-identify as being interested in the topics of brain tumors and hospice and/or palliative medicine (respectively) and may include patients, care partners, clinicians, researchers, advocates, and community health care organization members. #BTSM chat participants are typically brain tumor patients and care partners along with a few clinicians and researchers who specialize in brain tumors; #HPM attracts more clinician participants specializing in palliative care and hospice along with care partners and other advocates. Participants in the tweet chats analyzed here were not recruited and rather learned about the tweet chats through the hashtag communities’ regular promotional channels.

### Procedures

To engage diverse stakeholders in the tweet chats, we partnered with #BTSM and #HPM community leaders to promote the chats online through blog posts and email lists, and on social media via Twitter and Facebook Groups. The tweet chat hosts (@BTSMchat and @HPMchat, respectively) tweeted the 4 predefined topics (Table 1) with questions over a 60-minute period during a scheduled chat. The hosts alerted tweet chat participants that the transcript of the chat would be subject to qualitative analysis and used to inform research. One tweet question was posted roughly every 15 minutes. Twitter users responded to the questions and engaged in discussions with each other. On Twitter, responses are limited to 280 characters, and participants were instructed to add the #BTSM or #HPM hashtag to aggregate the conversation.

**Table 1.** #BTSM and #HPM Tweet Chat Topics

#BTSM (brain tumor social media) chat topics for April 8, 2018	
Topic 1	When you hear the phrase “quality of life,” what does that mean to you as a brain tumor patient, care partner, or health care professional? #BTSM
Topic 2	Has your health care team talked with you about quality of life? What did that look like, and what did that mean to you and your loved ones? #BTSM
Topic 3	How do your personal values (spiritual, religious, scientific, etc) factor into decisions about your health care? #BTSM
Topic 4	Given where you are now (eg, in treatment, posttreatment), what does a “good health care outcome” look like to you? #BTSM
**#**HPM (hospice and palliative medicine) chat topics for April 25, 2018	
Topic 1	(Part A) As a health care professional, how do you help patients make medical decisions when the person is suffering from a neurological/cognitive condition in which that person is no longer the person they used to be, or struggling to make decisions? #HPM
	(Part B) For anyone who isn’t working in health care: How does your health care team help when you find your way if you’re struggling to make medical decisions? #HPM
Topic 2	What kind of communication techniques work well for people suffering from cognitive difficulties? If you’ve experienced these conversations as a patient or family, do you have thoughts on what was good or not good about the health care professional’s approach? #HPM
Topic 3	Are there ever unintended consequences or unintended harm of navigating complex medical decisions and discussing palliative care with patients with neurological conditions? How can communication go wrong and how do you address those challenges? #HPM
Topic 4	How might we better improve on existing methods of discussing palliative care with patients living with serious neurological conditions? #HPM

Approximately 6 months after the tweet chats, we used Symplur^33^ (a social media analytics platform specializing in health care) to output into Microsoft Excel transcripts of the chats and available demographic information to describe participants. Symplur uses a proprietary algorithm that identifies and labels Twitter users by stakeholder perspective (eg, patient, clinician, researcher) and links participants’ locations from their public Twitter profiles. To generate a transcript a user enters the hashtag search term (eg, #BTSM or #HPM) and a date and time period for the search. For this analysis we searched #BTSM from 6 pm to 7 pm PT on April 8, 2018, and #HPM from 6 pm to 7 pm PT on April 25, 2018.

### Tweet Chat Topics

Tweet chat topics were first selected and written based on the interests of the BCQoLC, and were then refined in collaboration with @BTSMchat and @HPMchat organizers to ensure readability and consistency with Twitter standards (see Table 1). Given that the #HPM Twitter community includes health care professionals caring for patients with a variety of conditions other than brain tumors specifically, the #HPM chat topics focused on general QoL and decision making for people facing neurologic conditions.

### Qualitative Analysis

Qualitative content analysis^28^ of the tweet chat transcripts was used to identify themes related to how brain tumor stakeholders define QoL with brain tumors, and how neuro-oncology and palliative care clinicians communicate with patients and care partners about QoL, symptoms, disease progression, health care outcomes, and participation in research. Only *original tweets* were included in the analysis (*retweets* and *quoted tweets* were excluded to avoid “double counting” a result). The 2 chats were analyzed separately. Four researchers with qualitative analysis experience (B.M.K., H.D.L., G.V., M.G.) participated in the coding and thematic analysis process.

For each transcript, at least 2 of the 4 qualitative researchers independently read the transcripts and generated an inductive codebook with parent and child subcodes, including definitions of each code and the circumstances under which it should be applied. Next, each coder then independently coded his or her assigned transcript for the parent codes (eg, life experience, treatment experience) by indicating the presence of the code in each individual tweet in a column in Microsoft Excel. The coders met to compare coding, resolve discrepancies, and identify themes within and across each parent code. In February 2019, the research team re-engaged the #BTSM community in a discussion specifically about the QoL themes in a 1-hour open video chat for *member checking*, inviting them to comment and provide feedback on the credibility of the preliminary findings.^34^ The video chat was promoted in parallel with the scheduled #BTSM tweet chat in February 2019. The discussion focused on areas of the analysis #BTSM participants believed were lacking in clarity related to “communicating about and making decisions when there is uncertainty about the future of disease progression and treatment effects.”

## Results

### Demographics

Table 2 shows the participants in the #BTSM (N = 36 individuals, 417 tweets) and #HPM (N = 36 individuals; 355 tweets) chats by stakeholder type and geographic location. More than half (58%) of #BTSM chat participants comprised patients, advocates, and care partners. By comparison, more than half (61%) of #HPM chat participants comprised clinicians, researchers, and other health care professionals. The #BTSM chat saw participation from 3 neuro-oncology clinicians, and the #HPM chat was joined by 1 neuro-oncology clinician (who was also board certified in palliative care). Geographic data for 30 participants were obtained based on locations listed on public Twitter profiles.

**Table 2.** Demographics of 72 Tweet Chat Participants

	#BTSM chat	#HPM chat
Participant type^a^		
Patient	12	4
Care partner	3	2
Clinician	5 (3 neuro- oncology clinicians)	14 (1 neuro- oncology clinician)
Researcher	5	5
Other health care professional	2	3
Advocacy or health care organization	6	7
Unknown	3	1
Total	36	36
Total tweets (tweets per person)	417 (12)	355 (10)
Participant geography (data based on whether participant identifies geographic location in his or her public Twitter profile)		
Western US	2 (2 states: California, Colorado)	6 (3 states: California, Colorado, Nevada, Washington)
Southwestern US	1 (1 state: Arizona)	3 (1 state: Texas)
Midwestern US	4 (2 states: Kansas, Illinois)	3 (3 states: Kansas, Illinois, Ohio)
Northeastern US	3 (3 states: Connecticut, Massachusetts, New York)	2 (1 state: Pennsylvania)
Southeastern US	1 (1 state: North Carolina)	1 (1 state: Georgia)
Canada	4	0
Total participants identified by geography	15	15

Abbreviations: #BTSM, brain tumor social media; #HPM, hospice and palliative medicine; US, United States.

^a^Seven participants were common across the tweet chats, 3 of whom were members of the Brain Cancer Quality of Life Collaborative.

### Qualitative Themes from #BTSM Quality of Life Tweet Chat

Two major themes, and 7 minor themes, emerged from the #BTSM tweet chat of primarily patients with brain tumors and care partner stakeholders. The first major theme related to the many dimensions of what QoL means for patients and care partners. The second major theme related to how and when the health care system might address QoL when caring for people with brain tumors. See Supplementary Data 1 for a comprehensive set of illustrative quotes for each theme.

#### Major theme 1: Overall, quality of life for people with brain tumors and their care partners has many dimensions

—QoL is defined by the individual, may evolve over time, and is important for everyone—not just those with a terminal diagnosis or nearing the end of life. Participants cited a wide variety of concerns, including:

- physical functioning
- cognitive functioning and memory
- disease and treatment interfering with or “invading” life
- mood or spirits
- difficulty getting around and traveling
- overall suffering
- poor sleep
- nausea, constipation, pain, headaches
- seizures

Participants described psychosocial concerns, including:

- challenging behaviors resulting from effects of the tumor and treatment (noted by several care partners) such as personality changes
- irritable moods and violent outbursts
- changes in close relationships and in intimacy
- dependence on a broad social network and loss of independence and autonomy
- depression and anxiety
- financial toxicity
- grief over the loss of identity and things one previously enjoyed or was able to do

Desiring a sense of normalcy in daily life was common. As one brain tumor patient tweeted, *“QOL to me means keeping things as normal as possible for my children and my family, because stressing about them and worrying about the future does me no good, so we live in the now and enjoy life as much as possible.”*

##### Patients and Care Partners Experience Distress Due to Changes in Patients’ Sense of Self and Identity Due to the Effects of the Disease and its Treatment

Participants reflected on how brain tumors—depending on their location in the brain—and the surgical process of removal, compounded by side effects of chemotherapy and radiation, often affect physical and cognitive functioning as well as personality. For many, taking on a “sick role” and being dependent on others challenges the personal values for independence and self-sufficiency. The disease and its treatment can influence the ability to work and engage in hobbies, which for many is an important source of personal identity. A care partner explained, *“For [my husband], [quality of life] was working until the day before his final hospitalization—he was an architect and LOVED his work. And to keep traveling as much as we could.”*

##### A Good Health Care Outcome Is About More Than Being Alive or Having Stable Scans

Desirable outcomes include being alive and maintaining (or even improving) cognitive and physical functioning, feeling normal, feeling like yourself, and experiencing life and its milestones. A patient reported, *“In treatment, a good outcome would be regaining cognitive functioning, seeing the remaining tumor shrink, and having decades before it re-grows. Dream—a cure before it regrows.”*

##### Participants Described Surprise, Uncertainty, and Variability in the Experience of Brain Tumors, Which They Attributed in Part to Different Types of Tumors, Locations of Tumors, and the Variety of Treatments People Receive

Participants discussed feelings of surprise about not only the initial impact of the disease and treatment, but also changes over time. Patients and care partners noted feeling surprised about the onset of new symptoms, rapid decline, and how the disease and/or its treatment affected daily functioning. Changes sometimes happened quickly, almost overnight in some cases, and it took time to adjust. Patients and care partners mentioned lacking clear expectations for how the process would or could unfold, and articulated hindsight “if I had known at the beginning” types of statements. For instance, a care partner mentioned, *“I’m not even sure if it would have mattered, but I sometimes wish I better understood how things could so quickly change/destabilize/decline. It was very surprising….”*

#### Major theme 2: There is need to address quality of life in the context of health care, decision making about treatment, and support for care partners

##### —The Health Care System Needs to Provide Better Support for Care Partners

Participants described a need to address the burden to care partners and families, which potentially contributes to care partner burnout. A care partner emphasized that it is *“So important…to turn your head and look at the suffering caregiver sitting next to the patient and ask ‘How are you doing?’ Also ask caregiver lens on how the patient is doing because there may be forgetfulness or minimization. The tired caregiver knows what’s going on.”*

##### Patients and Care Partners Appreciate When Providers Discuss Quality of Life Early, but Not Immediately at Diagnosis, and Wish it Were Emphasized More

Patients and providers alike recognize that “quality of life” may not be a conversation that can or should happen right at diagnosis because patients and care partners will not or cannot hear it because they are often too overwhelmed with the diagnosis, information, and decisions to be made. A radiation oncologist acknowledged, *“It’s very difficult as a doctor to know exactly how much to be able to share/tell in the best way on the very first visit. Honesty is essential but so is tact and empathy. Individualizing isn’t easy.”* A tweet chat participant who is a physician, palliative care fellow, and care partner noted, *“Having these conversations as a daughter and a physician with my father was challenging, and I found it frustrating when he reported his doctors hadn’t talked to him about QoL. He and my mom didn’t hear it being discussed early on, and I wished they had.”*

##### Conversations About Quality of Life Emerge in the Context of the Patient-Provider Relationship, and Do Not Always Explicitly Use the Language “Quality of Life.”

Participants preferred when providers focused on developing a relationship with patients and care partners, which allowed discussions about QoL to emerge more organically. Tweet chat participants suggested that using the language “quality of life” is not always well received by patients, fearing it implies a transition to hospice care. Instead providers focus on getting to know a patient as an individual—his or her identity and values—and attend to the individual in decision making. A care partner explained, *“Even if we weren’t calling it QoL, we focused on being able to continue doing the things that mattered to us.”*

##### Patients Desire Access to Medical Journals and Scientific Evidence

One particular value that emerged for several patients concerned improved access to medical journals and scientific evidence so that people can do their own research to make their own decisions about what is best for them. One patient articulated, *“My personal values are more to the scientific side (from original training) = lists, research, data. Now [I] ask lots of questions to help me manage my health care. [I] need a flexible health care team to work with me on this.”*

### Palliative Care Communication Themes From #HPM Tweet Chat

The #HPM chat involving primarily hospice and palliative medicine clinicians, as well as other stakeholders (see Table 2), revealed similar themes to those from the #BTSM chat. We identified 6 themes. See Supplementary Data 1 for a comprehensive set of illustrative quotes for each theme.

#### Theme 3.1: Importance of early discussions about preferences for palliative care or hospice

—In parallel with the #BTSM themes, the #HPM community noted the importance of discussing preferences for palliative care and hospice care early. Because cognitive function and the ability for the individual to make decisions may change quickly, stakeholders emphasized discussing future care options early and explicitly. A clinician reported, ... *“as a primary care doc I am more assertive with frank talk about advance care planning and discussing wishes in patients with neurological conditions than any other. As soon as they’re diagnosed…”*

#### Preserving autonomy in palliative care decisions

—There is a fine line to balancing an individual’s autonomy with their reduced decision-making capacity due to cognitive impairments. Capacity and independence may ebb and flow over time for those with brain tumors rather than progress in one direction as in progressive neurological conditions. Preserving the individual’s autonomy in decisions about palliative care and hospice to the best extent possible is key. For instance, one clinician described, *“I try to sleuth out as clear a sense of their [the patient’s] whole personhood through those that know them well, pictures on the wall, magazines in the rack, and any actionable truths about themselves that they can share, even if garbled by illness.”* Preserving autonomy may involve relating to the patient on a human level, including using emotional or empathic language instead of technical language. A clinician shared that they tend to *“write things down, and repeat them. Consider hearing needs/difficulty. Consider shame the person is feeling. Consider the isolation they feel, as pt or caregiver.”*

#### Communication requires human connection and relationship

—As in the #BTSM chat, stakeholder perspectives in the #HPM chat emphasized the importance of human connection and relationship in communication related to palliative care and decision making. For example, a patient noted, *“Put the phone down. Close the computer. Sit down. Look me in the eye. Focus. Be present. Listen. Ask me to communicate what I understand. Listen. Recognize that I am a person, as a person I am bigger than my illness.”*

#### Communicate about expected changes (offer anticipatory guidance)

—In the context of the patient-clinician relationship, stakeholders noted that patients desire communication about what to expect regarding changes over time in how their neurological condition may affect their daily life and their independence. One participant noted, *“It’s funny you bring that up; my mom says the same: ‘No one told me he’d have to feed me.’ That’s when she wanted to know about her disease. What do you do when you lovecontrol?”*

#### Approach patient-family communication as a unit with independent parts

—Given the expected shift in independence and the patient’s capacity for decision making over time, while still preserving autonomy, participants suggested approaching the patient/care partner/family as a unit of independent parts. That is, cognitive changes necessitate carefully engaging in collective, as well as sequential, conversations with the patient and family members both together and individually. One palliative care physician shares this advice: *“If cognitive impairment leads to inability for deeper [goals of care] convo but patient is still contributing, I transparently tell pt that I’d like to talk to fam and why and ask pt what s/he would like us to keep in mind. Then ask what pt wants to know after I talk to fam.”*

#### Identify strategies to assess decision-making capacity

—#HPM chat participants noted the importance of ongoing assessment of decision-making capacity and discussed a variety of clinical strategies for assessing decision-making capacity, including asking other clinicians for suggestions. For example, *“I’m a [palliative care nurse] and where I am, capacity eval falls on the hospital psychologist and it’s all-or-nothing, so I was curious about alternatives!”* In response, clinicians shared a capacity assessment resource.^35^ Others noted their approach of using a “sliding capacity scale,” where it is incumbent on the clinician to recognize the nuances of assessing how cognitive impairment may be affecting one’s ability to make some decisions but not others.

## Discussion

This analysis contributes to the literature both on opportunities for improving QoL for brain tumor patients and care partners, and on methods for stakeholder engagement in research. In both tweet chats, patients, care partners, and clinicians emphasized that QoL is individual and evolving, and should be discussed earlier in the illness process. QoL concerns are ideally raised soon after but not right at diagnosis. These conversations require tact and empathy and are ideally embedded in trusting relationships. The themes that emerged in *both* the #BTSM chat (which had more patients) and the #HPM chat (which had more clinicians) emphasized that capacity and independence for those with brain tumors can ebb and flow over time rather than progress in one direction, as in progressive neurological conditions.

Addressing QoL requires understanding and respecting what QoL means for each individual to achieve (and maintain) goal-concordant care. A recent systematic review found that the use of QoL assessment tools are seldom used in brain cancer clinical practice, yet these tools may improve patient-provider communication and have the potential to improve care.^17^ Other research suggests that a variety of psychosocial and physical factors influences QoL in people with brain tumors, including challenging family dynamics, behavioral and mood disorders, poor emotional health, or physical impairments.^36^

Patients with brain tumors and care partners have myriad social, emotional, and spiritual needs, although few studies have comprehensively explored these concerns.^37^ Our findings echo previous stakeholder engagement efforts emphasizing the importance of autonomy and independence as contributing factors in QoL,^38^ as well as decision making. Other researchers^13^ have called for increased palliative care support for patients with brain tumors to address these physical, psychosocial, and spiritual needs. Research on palliative care services for neuro-oncology may consider the unique symptomatology (such as QoL concerns related to seizures and personality changes) of brain tumor patients.^39^

Finally, patients, care partners, and clinicians participating in these chats discussed the challenge of addressing surprise and uncertainty, which is important in all cancers, but may be especially valuable for those with brain tumors because the disease is often associated with rapidly changing needs and severe symptomatology. Research indicates that the informational needs of brain tumor patients and care partners vary greatly and include prognosis, symptom management, and treatment options.^40^ There are opportunities to study how to meet these needs with a shared decision-making approach with an interprofessional palliative care team—as has been shown to be effective in other domains for improving communication, reducing anxiety, and improving QoL.^41^ Providers may be reluctant to share distressing information, leading to a lack of prognostic awareness.^42,43^ When the patient’s health declines, patients and families may feel unprepared. Patients and care partners report a hunger for information, including about prognosis.^42,43^ This lack of effective information exchange may drive patients and families to seek access to scientific publications on their own, as evidenced by comments shared in the tweet chats.

Another goal of this work was to assess the extent to which Twitter would be an effective strategy for stakeholder engagement. We expected Twitter would aid in engaging diverse stakeholder perspectives from among the brain tumor and palliative care communities, overcome geographic barriers that limit the inclusion of individuals with rare diseases,^23^ and help stakeholders learn from each other. Themes identified in this analysis were consistent with each other and with the perspectives of the BCQoLC stakeholders. When themes parallel and align, it suggests concordance across chat groups and stakeholder types. Thus, Twitter was an effective strategy for corroborating stakeholder panel perspectives.

Twitter uses relatively few research resources compared to recruiting for and conducting in-person focus groups and can capture commonalities within a shared experience. A formal qualitative analysis, such as what was conducted in this paper, is not required to gain important insights from tweet chats.

There are limitations to using Twitter for stakeholder engagement. For example, we were unable to obtain detailed information about the participants such as age, sex, geographic setting (rural vs urban)—and unable to determine tumor type, histology, stage of disease, and other important clinical aspects for patient participants. Having more information about a patient’s diagnosis and history would help us better understand if the responses are relevant to a variety of tumor types. Finding ways to link tweet chat participants with other data sources to obtain demographic and medical history is an opportunity for future research. Additionally, tweet chats require collaboration with existing communities to be successfully attended and is limited to Twitter users, and thus the findings may not be generalizable to a larger population. An important consideration with tweet chats is that participants are likely those who are already familiar with these communities and open to sharing personal health information on a public forum (ie, introducing a selection bias), which may limit perspectives. Rigorous research is needed on comparative effectiveness of different engagement methods to assess differences in representative participation, experiences, and perspectives. For instance, different research priorities may emerge when engaging Twitter users compared to users of other social media platforms, or those who do not regularly use social media.

## Conclusion

This study demonstrated how social media–based methods of stakeholder engagement in research can inform research priorities, and can complement engagement using stakeholder panels. Innovations in engagement methods—such as the tweet chat method used in this paper—are still emerging. Not too long ago, the idea of involving patients and families in the design and conduct of neuro-oncology research may have been dismissed. Today, accepted engagement methods make it feasible and desirable for people of varying backgrounds and expertise to connect, collaborate, and co-create research.

Our engagement method used Twitter to inform opportunities for research on QoL and palliative care for people with brain tumors. According to our analysis, there is an opportunity for improving communication and services for people with brain tumors and their care partners to address a range of psychosocial and informational needs, as well as provide effective symptom management. The core principles of palliative care—with its collaborative team approach, exquisite attention to symptom management, and focus on expert communication—provide an avenue to address these unmet QoL needs. A brain tumor–specific palliative care model could be expanded to include an interprofessional care team (eg, physician, nurse, social worker, chaplain, rehabilitative services) and attention to providing care partner support and education. Informed by the results of our tweet chat analyses, our BCQoLC stakeholders have prioritized research on such a comprehensive, interprofessional model of palliative care that addresses a range of QoL needs for patients with brain tumors and their care partners.

## Supplementary material

Supplementary material is available online at *Neuro-Oncology Practice* (http://nop.oxfordjournals.org/).

npaa043_suppl_Supplemenatary_Data
