# Supplementary material for: Stakeholder engagement in research on quality of life and palliative care for brain tumors: a qualitative analysis of #BTSM and #HPM tweet chats
Source: Neurooncol Pract. 2020 Jul 29;7(6):676–84. doi: 10.1093/nop/npaa043 (PMC7716141; doi:10.1093/nop/npaa043)
Supplement: npaa043_suppl_Supplemenatary_Data [file npaa043_suppl_supplemenatary_data.docx]

**Supplementary Data 1. #BTSM & #HPM tweet chat themes and illustrative quotes**

| **Themes** | **Illustrative quotes*** |
| --- | --- |
| **#BTSM chat themes** | |
| **Theme 1: Quality of life for people with brain tumors and their care partners has many dimensions** | |
| Participants cited a wide variety of concerns including changes in physical functioning, cognitive functioning and memory, and psychosocial issues | *“QOL to me means keeping things as normal as possible for my children and my family, because stressing about them and worrying about the future does me no good, so we live in the now and enjoy life as much as possible.” – patient*  *“QoL for me = being able to do things in my life that make me happy and fulfilled. This can change over time.” – patient* |
| Patients and care partners experience distress due to changes in patients’ sense of self and identity due to the effects of the disease and its treatment | *“For {patient}, that was working until the day before his final hospitalization - he was an architect and LOVED his work. And to keep traveling as much as we could.” – care partner*  *“Because of motor deficits and seizures, I already have to rely on my mother more than I'd like. But it's important to me to be as independent as possible.” – patient* |
| A good healthcare outcome is about more than being alive or having stable scans | *“In treatment, a good outcome would be regaining cognitive functioning, seeing the remaining tumor shrink, and having decades before it re-grows. Dream - a cure before it regrows.” – patient*  *“A good healthcare outcome would mean being alive with as few as possible side effects and little disability.” — patient*  *“My current decisions are based on wanting to watch my kids grow up, but not have them see me suffer.” – patient* |
| Patients and care partners were surprised by symptoms and effects of treatment | *“Who can fathom such life devastation w/barely any time to adjust?! It is not Alzheimer's nor elderly expected decline. It is one day having your life blown up & QoL is how well you can pick up the pieces and reassemble some new strange configuration of a life.” – care partner*  *“I'm not even sure if it would have mattered, but I sometimes wish I better understood how things could so quickly change/destabilize/decline. It was very surprising…”* *– care partner* |
| **Theme 2: There is need to address quality of life in the context of healthcare, decision making about treatment, and support for care partners** | |
| The healthcare system needs to provide better support for care partners | *“So important… to turn your head and look at the suffering caregiver sitting next to the patient and ask How are you doing? Also ask caregiver lens on how the patient is doing because there may be forgetfulness or minimization. The tired caregiver knows what's going on.” – care partner* |
| Patients and care partners appreciate when providers discuss quality of life early, but not immediately at diagnosis, and wish it was emphasized more | *“I don't recall the phrase of ‘QOL’ came up specifically. But the side effects were clearly discussed pre-treatment. Having gone thru treatment, now I realized if #QOL was more emphasized to the patient, the process would have gone better.” – patient*  *“Having these conversations as a daughter and a physician with my father was challenging, and I found it frustrating when he reported his doctors hadn’t talked to him about QoL. He and my mom didn’t hear it being discussed early on, and I wished they had.” – physician, palliative care fellow, and care partner* |
| Conversations about quality of life emerge in the context of the patient-provider relationship, and don’t always explicitly use the language “quality of life” | *“Even if we weren't calling it QoL, we focused on being able to continue doing the things that mattered to us.” – care partner*  *“Our wonderful neuro-oncologist, so full of empathy... always in few words let us know he was/is by our side & there would be options along the way 2 consider. This helped QoL because it eased the terror... He could still smile w/us & chat w/my husband 2 give him dignity.”* – care partner  *“It's very difficult as a doctor to know exactly how much to be able to share/tell in the best way on the very first visit. Honesty is essential but so is tact and empathy. Individualizing isn't easy.” – radiation oncologist* |
| Patients desire for access to medical journals and scientific evidence | *“My personal values are more to the scientific side (from original training) = lists, research, data. Now [I] ask lots of questions to help me manage my health care. [I] need a flexible health care team to work with me on this.” – patient*  *“I value evidence-based practice and experience, thus, I do a whole heck of a lot of research before I make a decision. I researched and chose my neurosurgeon at the age of 17 after consulting medical journals, patient message boards, textbooks, etc.” – patient, who is now a medical social worker* |
| **#HPM chat themes** | |
| Early discussions about preferences for palliative care or hospice is important | *“... as a primary care doc I am more assertive with frank talk about advance care planning and discussing wishes in patients with neurological conditions than any other. As soon as they're diagnosed…” – clinician* |
| Preserving autonomy in palliative care decisions | *"I try to sleuth out as clear a sense of their [the patient's] whole personhood through those that know them well, pictures on the wall, magazines in the rack, and any actionable truths about themselves that they can share, even if garbled by illness." – clinician*  *“[I tend to] write things down, and repeat them. Consider hearing needs/difficulty. Consider shame the person is feeling. Consider the isolation they feel, as pt or caregiver." – clinician* |
| Communication requires human connection and relationship | *“Put the phone down. Close the computer. Sit down. Look me in the eye. Focus. Be present. Listen. Ask me to communicate what I understand. Listen. Recognize that I am a person, as a person I am bigger than my illness.” – patient* |
| Communicate about expected changes (offer anticipatory guidance) | *“It's funny you bring that up; my mom says the same. ‘No one told me he'd have to feed me.’ That's when she wanted to know about her disease. What do you do when you love control?” – care partner* |
| Approach patient-family communication as a unit with independent parts | *“If cognitive impairment leads to inability for deeper [goals of care] convo but patient is still contributing, I transparently tell pt that I’d like to talk to fam and why and ask pt what s/he would like us to keep in mind. Then ask what pt wants to know after I talk to fam” – palliative care physician* |
| Identify strategies to assess decision-making capacity | *“I'm a [palliative care nurse] and where I am, capacity eval falls on the hospital psychologist and it's all-or-nothing, so I was curious about alternatives!” – palliative care nurse* |

*Note: Individuals quoted granted the authors permission to included their tweets in this paper. Quotes selected were not by participating authors.*
